# MicroRNA Regulates Early-Life Stress–Induced Depressive Behavior via Serotonin Signaling in a Sex-Dependent Manner

**DOI:** 10.1192/j.eurpsy.2023.1268

**Published:** 2023-07-19

**Authors:** Y. Dwivedi

**Affiliations:** Psychiatry, University of Alabama at Birmingham, Birmingham, United States

## Abstract

**Introduction:**

The underlying neurobiology of early-life stress (ELS)-induced major depressive disorder (MDD) is not clearly understood. miRNAs, a subclass of noncoding RNAs, are estimated to regulate 20-90% of genes in the genome. Previous studies identify differences in miRNA expression following MDD and ELS, but there is limited research on a direct link between changes in serotonin signaling and miRNAs in response to ELS.

**Objectives:**

In this study, we used MS and environmental enrichment to study serotonin signaling in the PFC and its regulation by miRNAs. Because women are more likely to develop MDD, but there is no strong evidence of sex differences in the experience of ELS, we were interested to test for sex differences. We hypothesized that genes in the serotonin signaling pathway would be altered by ELS and potentially recovered by enrichment.

**Methods:**

We used maternal separation (MS) as a rodent model of ELS and tested whether microRNAs (miRNAs) target serotonin genes to regulate ELS-induced depression-like behavior and whether this effect is sex dependent. We also examined whether environmental enrichment prevents susceptibility to depression- and anxiety-like behavior following MS and whether enrichment effects are mediated through serotonin genes and their corresponding miRNAs.

**Results:**

MS decreased sucrose preference, which was reversed by enrichment. Males also exhibited greater changes in forced swim climbing and escape latency tests only following enrichment. *Slc6a4* and *Htr1a* were upregulated in the frontal cortex following MS. In male MS rats, enrichment slightly reversed *Htr1a* expression to levels similar to control rats. miR-200a-3p and miR-322-5p, which target *SLC6A4*, were decreased by MS, but not significantly. An *HTR1A*-targeting miRNA, miR-320-5p, was also downregulated by MS and showed slight reversal by enrichment in male animals. miR-320-5p targeting of *Htr1a* was validated in vitro using SHSY neuroblastoma cell lines.

**Conclusions:**

Altogether, this study implicates miRNA interaction with the serotonin pathway in ELS-induced susceptibility to depression-related reward deficits. Furthermore, because of its recovery by enrichment in males, miR-320 may represent a viable sex-specific target for reward-related deficits in major depressive disorder.

**Disclosure of Interest:**

None Declared

